# APLP1 as a cerebrospinal fluid biomarker for γ-secretase modulator treatment

**DOI:** 10.1186/s13195-015-0160-z

**Published:** 2015-12-22

**Authors:** Simon Sjödin, Kerstin K. A. Andersson, Marc Mercken, Henrik Zetterberg, Herman Borghys, Kaj Blennow, Erik Portelius

**Affiliations:** Institute of Neuroscience and Physiology, Department of Psychiatry and Neurochemistry, The Sahlgrenska Academy at the University of Gothenburg, Sahlgrenska University Hospital, Mölndal, S-431 80 Sweden; Janssen Research and Development, Beerse, Belgium; UCL Institute of Neurology, University College London, London, UK

## Abstract

**Introduction:**

Alzheimer’s disease brains are characterized by extracellular plaques containing the aggregated amyloid β_42_ (Aβ_42_) peptide and intraneuronal tangles containing hyperphosphorylated tau. Aβ_42_ is produced by sequential processing of the amyloid precursor protein (APP) by β-secretase followed by γ-secretase. Substantial efforts have been put into developing pharmaceuticals preventing the production or increasing the clearance of Aβ_42_. However, treatments inhibiting γ-secretase have proven disappointing due to off-target effects. To circumvent these effects, γ-secretase modulators (GSMs) have been developed, which rather than inhibiting γ-secretase shift its preference into producing less aggregation-prone shorter Aβ peptides. Belonging to the same family of proteins as APP, amyloid-like protein 1 (APLP1) is also a substrate for γ-secretase. Herein we investigated whether the GSM E2012 affects APLP1 processing in the central nervous system by measuring APLP1 peptide levels in cerebrospinal fluid (CSF) before and after E2012 treatment in dogs.

**Methods:**

An in-house monoclonal APLP1 antibody, AP1, was produced and utilized for immunopurification of APLP1 from human and dog CSF in a hybrid immuno-affinity mass spectrometric method. Seven dogs received a single dose of 20 or 80 mg/kg of E2012 in a randomized cross-over design and CSF was collected prior to and 4, 8 and 24 hours after dosing.

**Results:**

We have identified 14 CSF APLP1 peptides in humans and 12 CSF APLP1 peptides in dogs. Of these, seven were reproducibly detectable in dogs who received E2012. We found a dose-dependent relative increase of the CSF peptides APLP1β17, 1β18 and 1β28 accompanied with a decrease of 1β25 and 1β27 in response to E2012 treatment. All peptides reverted to baseline over the time of sample collection.

**Conclusion:**

We show an in vivo effect of the GSM E2012 on the processing of APLP1 which is measurable in CSF. These data suggest that APLP1 peptides may be used as biomarkers to monitor drug effects of GSMs on γ-secretase processing in clinical trials. However, this requires further investigation in larger cohorts, including studies in man.

## Introduction

Alzheimer’s disease (AD) is a progressive neurodegenerative disorder and the most prevalent form of dementia [1]. It is characterized by extracellular plaques, containing aggregated amyloid-β (Aβ) peptides [2], and intraneuronal tangles consisting of hyperphosphorylated tau [3]. In the amyloid cascade hypothesis, it is postulated that there is an imbalance in the production and/or clearance of Aβ which is believed to cause a series of events including microglial activation, oxidative stress, neuronal dysfunction, formation of tangles and inevitable neurodegeneration [4]. For these reasons, targeting the production of the aggregation prone and potentially toxic 42 amino acid residue-long variant of Aβ (Aβ_42_) has been subject to extensive research with the aim of developing disease modifying compounds that inhibits or modulates the enzymes responsible for the formation of Aβ (see elaborating reviews elsewhere [5–7]).

The amyloid precursor protein (APP) is subjected to proteolytic processing by three secretases: α-secretase, β-secretase and γ-secretase. The proteolytic activity of α-secretase is likely attributed to the protein disintegrin and metalloproteinase domain-containing protein 10 (ADAM10) [8]; β-secretase has been identified as β-site APP-cleaving enzyme (BACE) [9]; whereas γ-secretase is a multisubunit protein complex consisting of nicastrin (NCSTN), anterior pharynx-defective 1 (APH-1), presenilin enhancer 2 (PEN2) and the N- and C-terminal fragments of presenilin 1 or 2 (PS1 or PS2) [10]. Aβ_42_ is produced through the amyloidogenic pathway where APP is cleaved by β-secretase [9] and subsequently by γ-secretase [11]. Alternatively Aβ_42_ production is prevented in the non-amyloidogenic pathway where α-secretase rather than β-secretase cleaves APP within the Aβ sequence generating shorter Aβ peptides [12].

Secretases are an appealing target to alter the metabolism of Aβ. However, recent clinical trials where AD patients were treated with γ-secretase inhibitors failed to reach their primary clinical endpoints; the cognitive decline was even worse in the treatment arm than in placebo [13, 14]. A diverse array of transmembrane proteins have been identified as γ-secretase substrates [15], including Notch [16, 17], and the problems associated with γ-secretase inhibitor treatment may be explained by the physiological functions of these substrates [15].

To overcome the possible negative effects of inhibiting γ-secretase, γ-secretase modulators (GSMs) have been developed to shift the production from the amyloidogenic Aβ_42_ to less aggregation-prone peptides (e.g*.*, Aβ_38_) without affecting the release of the intracellular domains of APP and other γ-secretase substrates such as Notch1, cadherins, Erb4 and EphB2 [18]. Thus, these compounds modulate the cleavage pattern of Aβ without inhibiting, for example, Notch signaling. The effect of GSMs on APP processing has previously been shown to be measurable in vivo in cerebrospinal fluid (CSF) from, for example, monkeys [19] and guinea pigs [20], where the level of Aβ_42_ decreased and Aβ_37_ or Aβ_38_ increased [19, 20]. In agreement with these studies, we recently showed that dogs treated with the GSM E2012 displayed a distinct shift in the CSF Aβ peptide pattern with decreased levels of Aβ_42_ as well as Aβ_39_ and Aβ_40_ accompanied with increased levels of Aβ_37_ [21]. The effects of different GSMs on Aβ production have been summarized in a review by Crump et al. [22].

Amyloid-like protein 1 (APLP1) and APP share, to a large extent, similar structural domains and are both suggested to be involved in neurite outgrowth, cell adhesion and neuronal migration [23]. APLP1 and APP are also known to form heterodimers with possible implications in cell adhesion and synaptogenesis [24, 25]. APLP1 has been suggested to be processed by the same enzymatic activities as APP including γ-secretase and α-secretase, generating a p3-like peptide called ALP-1 [24, 26], similar to the putative p3 fragment identified as a possible proteolytic product from APP [27]. While it is established that γ-secretase is responsible for the C-terminal processing of APLP1 [28–30], the secretase cleaving at the N-terminus has been suggested to be either α- [26] or β-secretase [28, 31].

Recently, it was shown that APLP is processed into short Aβ-like peptides (APLP1β25, 1β27 and 1β28) [28] and that the concentrations of CSF APLP1β25 and 1β27 were decreased while the concentration of CSF APLP1β28 was unchanged in four different PS1 mutation carriers [32] leading to an increased APLP1β28 to total APLP1β ratio [28]. In addition, cells treated with the Aβ_42_ raising GSM S2474, or cells harboring certain PS1 mutations were also found to have an increased APLP1β28 to total APLP1β ratio and it was suggested that this ratio may reflect the Aβ_42_ production in the brain [28]. However, how the S2474 treatment or PS1 mutations affected the APLP1β28 concentrations were not reported. Whether APLP1 peptides can be used as CSF AD biomarkers reflecting Aβ_42_ production needs to be investigated in large clinical studies, including early clinical stage of AD, AD dementia and control subjects, in which other CSF and imaging biomarkers are included.

In order to characterize APLP1 in CSF, we developed a monoclonal antibody directed against APLP1. We then used hybrid immuno-affinity-based enrichment of CSF APLP1 peptides from dogs followed by mass spectrometry (MS) analysis. In the present study we tested the hypothesis that peptides derived from APLP1 can be used as in vivo biomarkers reflecting treatment with the second generation GSM E2012.

## Methods

### Chemicals

The γ-secretase modulator E2012 (cas no. 870843-42-8, (3E)-1-[(1*S*)-1-(4-fluorophenyl)ethyl]-3-[[3-methoxy-4-(4-methyl-1H-imidazol-1-yl)phenyl]methylene]-2-piperidinone) was synthesized according to the described procedure [33].

### Experimental animals

Beagle dogs were given a single oral dose of 20 and 80 mg/kg E2012 with a dosing interval of at least 1 week. The animals were exposed in a randomized cross-over study as previously described [21]. The animals (seven males, age 6–8 years) received the compound with a liquid meal between 7 and 8 am and 1 ml CSF was collected prior to the meal and 4, 8 and 24 hours after dosing. The meal consisted of 120 ml concentrated liquid (Convalescence support, one satchel dissolved in 112.5 ml warm water; Crown Pet Foods Ltd., Castle Cary, UK) to which the compounds had been added directly before feeding. CSF was collected from awake animals from the lateral ventricle via a cannula. The CSF was collected into polypropylene tubes, immediately placed on dry ice and stored at −80 °C until analysis. The method for implanting and collecting CSF via the cannula in dogs has been described elsewhere [34]. In brief, a hole was drilled in the cranium of the dogs into which the cannula was screwed. The cannula was positioned at the following coordinates: 38–40 mm rostral to the occipital protuberance and 8 mm lateral to the center of the sagittal crest. The study was conducted according to protocol 2009-252-SD MECHPHA, approved by the Ethical Committee on Laboratory Animal Testing (Ethische Commissie Dierproeven, Johnson & Johnson Pharmaceutical Research and Development, Beerse, Belgium).

### Generation of anti-APLP1 antibody

A monoclonal antibody against APLP1, named AP1, was generated by immunization of 8-week-old Balb/c mice with the KLH-conjugated peptide APLP1 (amino acid 568–579, [UniProtKB:P51693]; Caslo ApS, Lyngby, Denmark) in complete Freund’s adjuvant (Sigma-Aldrich Co., St Louis, MO, USA). After three dosages (approximately 75 μg/mouse), the spleen was removed and B cells were fused with the myeloma cell line SP2/0 following standard procedure. Approximately 10 days after fusion, cell media were screened for APLP1 antibodies using APLP1 full length and neurogranin (Caslo ApS) as a negative control. Clones which reacted with the APLP1 protein were further grown, subcloned and subsequently frozen in liquid nitrogen. The isotype was determined using a commercially available kit (Pierce Rapid Isotyping Kit-Mouse; Thermo Fisher Scientific Inc., Waltham, MA, USA). Finally, antibodies were purified using a protein G column (GE Healthcare Bio-Sciences AB, Uppsala, Sweden).

### Immuno-affinity mass spectrometry

CSF collected prior to dosing and 4, 8 and 24 hours after dosing with 20 or 80 mg/kg E2012 was analyzed by hybrid immuno-affinity MS as described previously with some modifications [21]. In brief, 4 μg of the in-house developed mouse monoclonal anti-APLP1 antibody AP1 was incubated with 25 μl magnetic beads conjugated to sheep anti-mouse IgG (Dynabeads M280, Invitrogen, Thermo Fisher Scientific Inc.). Beads with the AP1 antibody complex were then washed in phosphate-buffered saline and added to 600 μl CSF containing 0.0025 % Tween, to a total volume of 625 μl. CSF with added beads was incubated over night at +8 °C on a rocking platform. Elution of APLP1 was performed in a five-step procedure using a KingFisher mL Magnetic Particle Processors system (Thermo Fisher Scientific Inc.). The eluate was dried and redissolved in 5 μl 0.1 % formic acid in 20 % acetonitrile. The sample in solution was mixed in a 2:1 ratio with a sample matrix containing 15 g/l alpha-cyano-4-hydroxycinnamic acid and 0.1 % trifluoroacetic acid in acetonitrile and spotted on an MTP 384 target plate polished steel TF (Bruker Daltonics GmbH, Bremen, Germany) with a seed layer containing 20 g/l alpha-cyano-4-hydroxycinnamic acid in 10 % methanol and 90 % acetone. The samples were analyzed as duplicates by matrix-assisted laser desorption ionization time-of-flight/time-of-flight (MALDI-TOF/TOF) using an UltrafleXtreme (Bruker Daltonics GmbH) mass spectrometer operating at positive reflector mode. The sample spectra acquired were processed using flexAnalysis v3.3 (Bruker Daltonics GmbH) by performing baseline subtraction, smoothening and internal calibration based on the theoretical monoisotopic masses of APLP1 fragments 1β17 (1615.79 *m/z*), 1β18 (1728.88 *m/z*), 1β22 (2143.11 *m/z*), 1β25 (2328.19 *m/z*), 1β27 (2472.24 *m/z*) and 1β28 (2585.32 *m/z*). Exported peak lists were subjected to processing by an in-house MATLAB (Mathworks Inc., Natick, Massachusetts, USA) program which, in short, relative to the monoisotopic peak, integrated the peak areas within the limits of −2 to +5 *m/z*. Following this, the integrated peak areas in each specific spectrum were normalized as the percentage of the total area of all peaks measured. Duplicate measurements were averaged and then a fold-change was calculated for samples acquired at 4, 8 and 24 hours against the sample collected prior to dosing as baseline.

### Tandem mass spectrometric identification of peptides

Employing collision-induced dissociation and the LIFT technology of the UltrafleXtreme, tandem MS (MS/MS) spectra of selected APLP1 peptides were acquired. These were processed and annotated in the BioTools software v3.2 (Bruker Daltonics GmbH) by employing an in-house Mascot Deamon server (v2.3.2; Matrix Science Ltd. London, UK) search against an in-house designated APLP1 [UniProtKB:P51693] database. The parameters for searching the database were set to singly charged ions, variable methionine oxidation, a mass tolerance of 200 ppm, MS/MS tolerance of 0.5 Da and a MALDI-TOF/TOF instrumentation. MS and MS/MS spectra were also acquired from human CSF using the same sample preparation methodology as described above. CSF samples were supplied by clinical routine at the Clinical Neurochemistry Laboratory, The Sahlgrenska University Hospital, Mölndal, Sweden, in accordance with ethical approval from the regional ethics committee at the University of Gothenburg. The samples were de-identified by pooling.

### Statistical analysis

Statistical analysis was performed using GraphPad PRISM v6.04 (GraphPad Software, Inc., San Diego, USA) comparing the fold-change within each of the doses for the indicated peptides with Friedman test and employing Dunn’s test for correction of multiple comparisons. Significant results were considered differences with a calculated *p*-value of <0.05.

## Results

### Identification of APLP1 peptides in CSF

By combing the selectivity of the in-house mouse monoclonal antibody, AP1, and MS, we here for the first time show that several APLP1 derived peptides may be used as biomarkers for target engagement of the GSM E2012 in vivo.

**Fig. 1:**
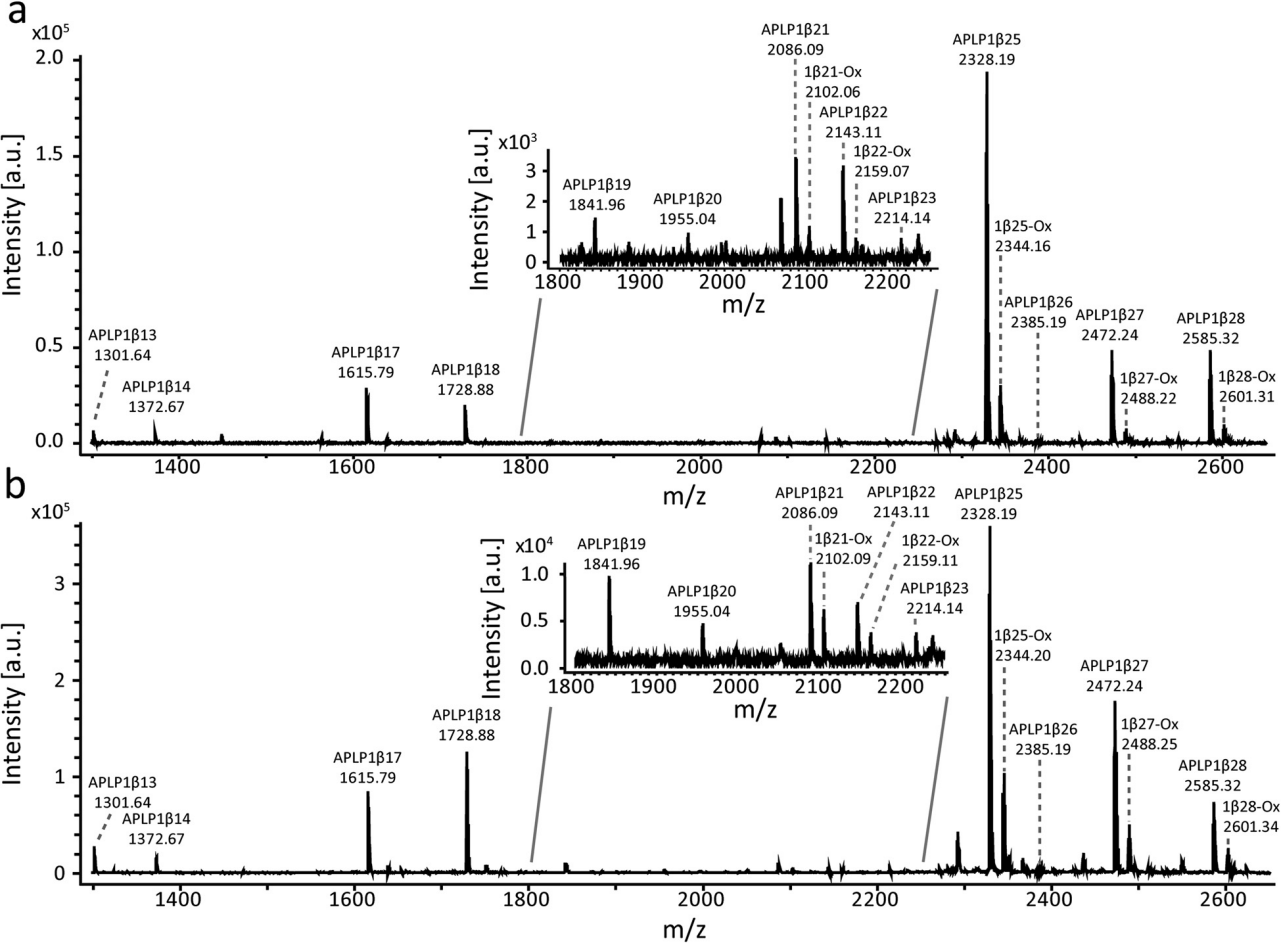
Full mass spectrum of APLP1 immunoprecipitated from cerebrospinal fluid. APLP1 was immunoprecipitated from **a** dog or **b** human cerebrospinal fluid and subjected to MALDI-TOF/TOF in which a full mass spectrum was recorded. The peaks corresponding to the APLP1 peptides that have been identified by their corresponding theoretical masses or tandem mass spectrometry are annotated in the spectrum. The insert shows a magnification of the peaks detected between 1800 and 2250 *m/z*. Additional peaks of +16 *m/z* following APLP1β21 to 1β28 corresponds to oxidation of methionine at position 21. *APLP1* Amyloid-like protein 1

**Fig. 2:**
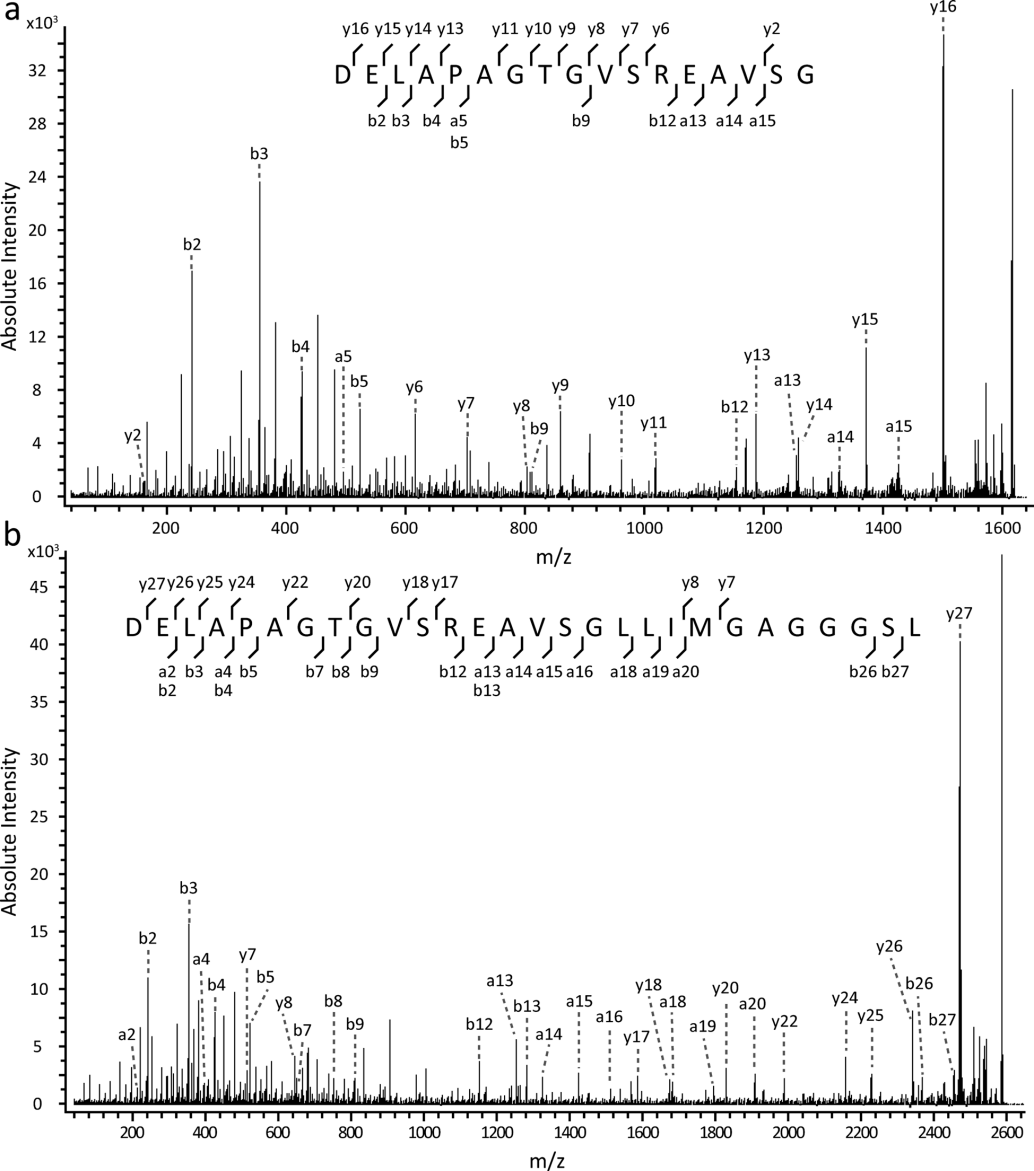
Fragment ion mass spectra of APLP1 peptides. Fragment mass spectra generated by tandem mass spectrometry of the peptides **a** APLP1β17 and **b** APLP1β28 immunoprecipitated from human cerebrospinal fluid. Shown are the fragment ions annotated as a, b and y ions. The fragment spectra were used for identification and annotation by searching an in-house designated APLP1 database using a Mascot Daemon server

The amino acid sequence for APLP1 for *Canis familiaris* and *Homo sapiens* has a 100 % homology [UniprotKB:J9JHP8 versus UniprotKB:P51693]. In total, 14 APLP1 peptides were identified in human CSF and 12 in dog CSF of which nine were confirmed by MS/MS (see Fig. 1 and Tables 1 and 2 for all identified peptides). All confirmed peptides started with the aspartic acid found at amino acid 568 of APLP1 [UniprotKB:P51693] and included peptides ranging between APLP1β13 up to APLP1β28. An additional peak that had the mass of the peptide plus 16 Da, corresponding to oxidation of methionine at position 21, was also observed for several of the APLP peptides (Fig. 1). Fragment ion mass spectra used for the identification of APLP1β17 and APLP1β28 are shown in Fig. 2a and b, respectively.

**Table 1:** APLP1 precursor ion identification in full mass spectrum

Sequence	Theoretical mass (Da)	Observed mass (Da) dogs	ppm dogs	Observed mass (Da) human	ppm human	Annotation
DELAPAGTGVSRE	1300.63	1300.60	−172	1300.63	39	APLP1β13
DELAPAGTGVSREA	1371.66	1371.65	−114	1371.67	20	APLP1β14
DELAPAGTGVSREAVSG	1614.78	1614.78	−8.5	1614.78	−0.058	APLP1β17
DELAPAGTGVSREAVSGL	1727.87	1727.87	13	1727.87	0.12	APLP1β18
DELAPAGTGVSREAVSGLL	1840.95	1840.96	12	1840.95	−8.2	APLP1β19
DELAPAGTGVSREAVSGLLI	1954.04	1954.04	8.5	1954.04	−8.5	APLP1β20
DELAPAGTGVSREAVSGLLIM	2085.08	2085.08	−2.4	2085.08	1.1	APLP1β21
DELAPAGTGVSREAVSGLLIMG	2142.10	2142.10	−7.6	2142.10	−0.46	APLP1β22
DELAPAGTGVSREAVSGLLIMGA	2213.14	–	–	2213.13	−13	APLP1β23
DELAPAGTGVSREAVSGLLIMGAG	2270.16	–	–	2270.16	8.5	APLP1β24
DELAPAGTGVSREAVSGLLIMGAGG	2327.18	2327.18	3.3	2327.18	1.0	APLP1β25
DELAPAGTGVSREAVSGLLIMGAGGG	2384.20	2384.19	−33	2384.19	−65	APLP1β26
DELAPAGTGVSREAVSGLLIMGAGGGS	2471.23	2471.23	−0.53	2471.23	−0.91	APLP1β27
DELAPAGTGVSREAVSGLLIMGAGGGSL	2584.32	2584.32	0.72	2584.32	0.31	APLP1β28

Full mass spectra of amyloid-like protein 1 (APLP1) immunoprecipitated from dog and human cerebrospinal fluid were acquired by MALDI-TOF/TOF. The Table shows the amino acid sequences, the observed masses in Da in dogs and human and their deviation from the theoretical peptide mass in ppm for the peptides identified

**Table 2:** Tandem mass spectrometric identification of APLP1 peptides

Peptide	Observed *m/z*	ppm	Ion score	Expect	a/b/y ions
APLP1β14	1372.71	30.4	48	1.6x10^-5^	–/4/6
APLP1β17	1615.83	23.7	79	1.3x10^-8^	4/6/11
APLP1β18	1728.91	21.9	65	3.0x10^-7^	–/8/10
APLP1β19	1842.06	56.7	18	1.7x10^-2^	–/5/4
APLP1β21	2086.19	48.1	24	3.9x10^-3^	–/6/–
APLP1β23	2214.14	−2.91	15	3.3x10^-2^	–/4/–
APLP1β25	2328.51	138	79	1.3x10^-8^	7/6/11
APLP1β27	2472.31	27.8	100	9.4x10^-11^	9/5/9
APLP1β28	2585.40	28.3	93	5.3x10^-10^	9/11/10

The Table shows the peptides that were identified by tandem mass spectrometry. The acquired fragment mass spectra were used for searching an in-house Mascot Deamon server containing a designated amyloid-like protein 1 (APLP1) database. Also shown are the observed masses and the deviation from the theoretical masses in ppm, as well as identification statistics given as the Mascot Ion Score and Expect value. The number of identified a, b and y ions are also indicated

### Modulation of APLP1 processing by E2012 in dogs

**Fig. 3:**
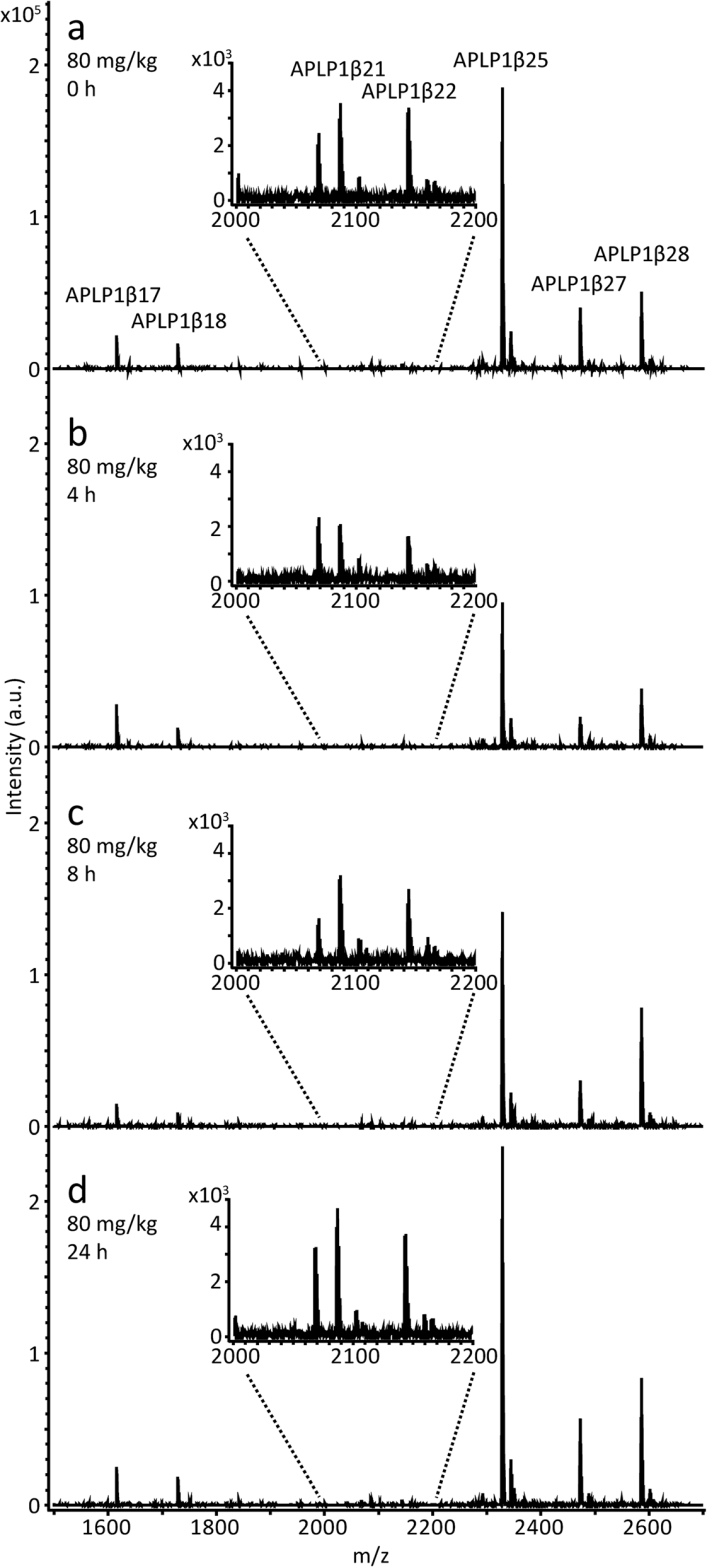
Full mass spectrum of 80 mg/kg γ-secretase modulation effect in dogs. Full mass spectra of APLP1 were acquired by hybrid immuno-affinity MALDI-TOF/TOF mass spectrometry. The spectra show the relative changes of the APLP1 peptides in cerebrospinal fluid collected from a dog **a** prior to dosing with 80 mg/kg of the γ-secretase modulator E2012 and subsequently **b** 4, **c** 8 and **d** 24 hours after dosing. *APLP1* Amyloid-like protein 1

In total, seven APLP1 peptides were reproducibly detected in all dog CSF samples (APLP1β17, 1β18, 1β21, 1β22, 1β25, 1β27 and 1β28) and their response to treatment with E2012 was further investigated. See Fig. 3 for representative mass spectra from a dog treated with 80 mg/kg of the GSM E2012.

**Fig. 4:**
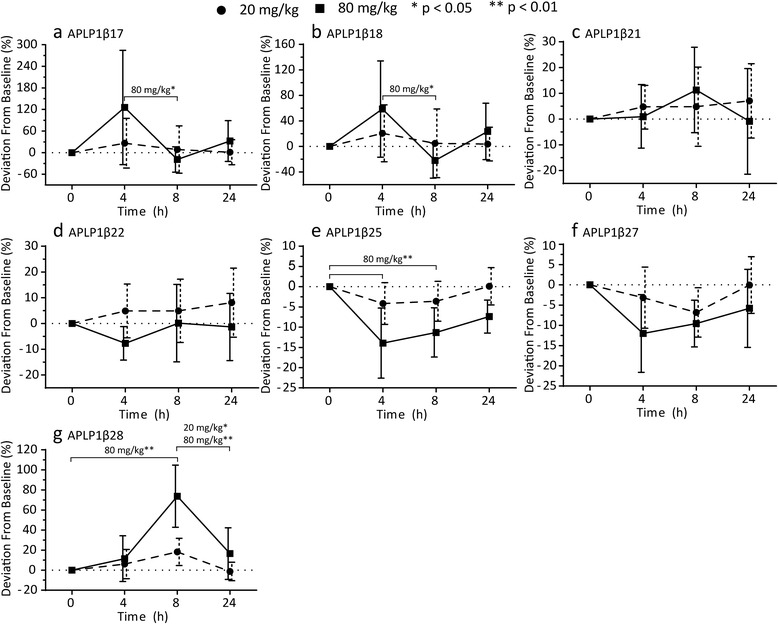
Modulation of γ-secretase processing of APLP1 in dogs. APLP1 was immunoprecipitated from cerebrospinal fluid collected from dogs prior to and 4, 8 and 24 hours after receiving 20 and 80 mg/kg of the γ-secretase modulator E2012. Using MALDI-TOF/TOF, full mass spectra were recorded in which the intensity of the peak corresponding to a peptide was normalized against the intensity of all the peaks measured in the spectrum. Shown is the percent deviation from the sample collected prior to dosing (baseline) for the peptides **a** APLP1β17, **b** APLP1β18, **c** APLP1β21, **d** APLP1β22, **e** APLP1β25, **f** APLP1β27 and **g** APLP1β28. The measurements are shown as the mean (n = 7) with 95 % confidence interval. Significant dose effects between time intervals were determined using Friedman test combined with Dunn’s test for multiple comparisons and are indicated as *p < 0.05 and **p < 0.01. *APLP1* Amyloid-like protein 1

Four hours after administration of the GSM E2012 (80 mg/kg), we found higher levels of APLP1β17 (*p* = 0.027) and APLP1β18 (*p* = 0.016) compared to 8 hours post-treatment. The increases reverted to baseline levels at 8 hours post-treatment and remained at baseline levels 24 hours post-treatment (Fig. 4a and b). No significant differences were observed for the two peptides after the 20 mg/kg dose (Fig. 4a and b). After administration of 80 mg/kg, the levels of APLP1β25 were significantly decreased both at 4 hours (*p* = 0.0032) and 8 hours (*p* = 0.0032) post-treatment (Fig. 4e). The 20 mg/kg dose showed a similar trend without reaching statistical significance (Fig. 4e). APLP1β28 showed a dose-dependent increase at 8 hours post-treatment which was significant for 80 mg/kg (*p* = 0.0032) but not 20 mg/kg (Fig. 4g). However, the increase at 8 hours was significant relative to 24 hours post-treatment for both doses (*p* = 0.0032 and *p* = 0.047 for 80 mg/kg and 20 mg/kg, respectively; Fig. 4g).

APLP1β21 and APLP1β22 did not show any significant deviations from baseline for either of the two doses (Fig. 4c and d). The level of APLP1β27 showed a trend towards decreased levels in a dose-dependent manner in response to treatment but it did not reach statistical significance for either of the doses (Fig. 4f).

## Discussion

In the present study, we show that APLP1 in CSF is present as a variety of short peptides. For the first time we also demonstrate that the levels of several CSF APLP1 peptides are markedly affected in response to treatment with the GSM E2012 suggesting that APLP1-derived peptides may be used as biomarkers to prove target engagement for this type of treatments. To our knowledge there are no studies of the effect of γ-secretase modulators on APLP1 processing in vivo to date.

Previously, using an explorative proteomics approach, we identified eight endogenous APLP1 peptides in human CSF including APLP1β17, 1β18, 1β25 and 1β27 [35]. By employing the targeted approach described herein we have identified and confirmed the presence of the previously identified peptides in CSF [28, 35] and have now extended the list of identified endogenous APLP1 peptides. A previous report showed an increase of APLP1β28 relative to the total amount of APLP1 peptides in CSF from AD subjects [28] while we recently showed that the levels of APLP1β25, 1β27 and 1β28 in CSF from Down’s syndrome patients are decreased as compared to healthy controls [36]. However, whether CSF APLP1β28 can be used as an AD or Down’s syndrome biomarker needs to be investigated in large clinical studies.

Of the 12 identified APLP1 peptides in dog CSF, we show that several are affected in response to treatment with the GSM E2012 and that some peptides are affected in a dose-dependent manner as the effect was more pronounced with the higher dose (80 mg/kg). We found increased relative levels of APLP1β17, 1β18 and 1β28 in response to treatment accompanied by decreased relative levels of APLP1β25 and 1β27. The changes in the levels for all of the peptides seem to revert towards baseline levels over time. For APLP1β21 and 1β22 we found no significant difference. These findings suggest that the modulation of the APLP1 processing affects the production over the range of peptides present, not only a single peptide or exclusively shorter or longer peptides. Furthermore, the transmembrane region of APLP1 ranges from position 581–603. Thus all identified APLP1 peptides, except APLP1β13, are located within this region and are accessible for γ-secretase processing.

Previously we have shown that the CSF levels of Aβ_37_ increases and Aβ_42_ decreases in dogs treated with the GSM E2012 [21]. Here we show in the very same material that the levels of several APLP1 peptides are affected in response to E2012 treatment. Thus investigating the level of APLP1 peptides in GSM treatment trials may be highly informative and possibly increase our understanding of the mechanistics and dynamics of APLP processing in the disease.

One limitation with the current study is that the epitope of the APLP1 antibody used (AP1) has not been determined. However MS analysis, including MS/MS, confirms that the peptides studied in the present study indeed are APLP1 derived. It should also be noted that the ratio between the different endogenous APLP1 peptides detected cannot be interpreted as a direct reflection of their absolute abundance in the CSF. This is because the ionization efficiency might be different for the different peptides. Phase I trials in man are needed to further study the potential of CSF APLP1 peptides to verify target engagement of GSM drug candidates as well as long-term clinical trials to examine if these biomarkers also may predict a beneficial clinical treatment effect.

With GSMs, one of the benefits is to avoid the potential inhibition of processing of non-intended substrates such as Notch [16, 17] which has been one of the main concerns when using γ-secretase inhibitors. However, GSMs have also shown an effect on Notch processing but in respect to cleavage site preference and not inhibition [37]. How GSMs affect the processing of the substrates is to a large extent unclear. It is likely dependent on the particular modulator used and the target with which it interacts [22]. First generation GSMs have been hypothesized to interact with the substrate, the secretase, or both [22], and it has been further speculated that, in the instance of modulators targeting the APP and Aβ sequences, this might affect the processing by adjusting the position of the amino acid sequence in relation to the membrane [38]. Second-generation GSMs, to which E2012 belongs, have been shown rather to interact with the subunits of γ-secretase [22]. Specifically, E2012 has been indicated to bind to the N-terminal fragment of PS1 in a competitive manner [39, 40].

## Conclusions

We have identified a number of APLP1 peptides in human and dog CSF. Furthermore we have shown that γ-secretase modulation, using the GSM E2012, affects APLP1 proteolytic processing in vivo and that this is reflected in the relative levels of a number of APLP1 peptides. The soluble nature of the APLP1 peptides [24, 28] and the fact that APLP1 is a γ-secretase substrate, as indicated herein and shown elsewhere [28–30], suggests that secreted APLP1 fragments may be informative when investigating γ-secretase cleavage products in AD. Our findings show that APLP1 peptides can be used as in vivo biomarkers for E2012 treatment, which may be translated to other second-generation GSMs.

## Abbreviations

Aβ: Amyloid β
AD: Alzheimer’s disease
APLP1: Amyloid-like protein 1
APP: Amyloid precursor protein
CSF: Cerebrospinal fluid
GSM: γ-Secretase modulator
MALDI-TOF/TOF: Matrix-assisted laser desorption ionization time-of-flight/time-of-flight
MS: Mass spectrometry
MS/MS: Tandem mass spectrometry
PS: Presenilin
